# Hot tongue on FDG PET scan in a patient of Hodgkin’s lymphoma undergoing antipsychotic treatment

**DOI:** 10.4103/0972-3919.72690

**Published:** 2010

**Authors:** Alok S Pawaskar, Hrishikesh Aurangabadkar, M Indirani, S Shelley

**Affiliations:** Department of Nuclear Medicine and PET-CT, Apollo Hospitals, Chennai - 600 006, India

**Keywords:** FDG PET, hot tongue, tardive dyskinesia. tongue uptake

## Abstract

Fluorine-18 fluorodeoxyglucose positron emission tomography-computed tomography (F-18 FDG PET-CT) is the modality of choice for the diagnosis, staging, and restaging of many malignancies. The importance of eliminating false positives cannot be underestimated because they can dramatically alter the clinical course. We present a case of benign uptake in the tongue secondary to tardive dyskinesia in a 53-year-old woman referred for therapy response evaluation of Hodgkin’s lymphoma who was concurrently receiving oral antipsychotic therapy. This case emphasizes the importance of detailed clinical history and examination when concluding definite diagnosis.

## INTRODUCTION

As use of fluorodeoxyglucose positron emission tomography (FDG PET) expands with increasing number of cases performed than ever, the population studied includes a wide spectrum of clinical conditions. With that, newer patterns of normal and abnormal FDG uptake are being recognized. It is very important to know the normal variants as well as the false-positive findings in a FDG PET study, as this modality is proven to be highly sensitive but with poor stand-alone specificity. Although addition of computed tomography (CT) to PET has helped to improve the specificity to some extent, some areas still present a diagnostic dilemma. Some of these puzzles can certainly be solved if we are careful enough to take a detailed clinical history and perform a thorough clinical examination.

We herein present an interesting case of intense FDG uptake in the tongue in a case of Hodgkin’s lymphoma that remained a mystery till the clinical examination brought to notice involuntary tongue movements and probing patient further revealed history of antipsychotic medications to explain the scan findings to our satisfaction.

## CASE REPORT

An apparently healthy 53-year-old lady of Indian origin presented to our hospital with history of high-grade fever and itching all over the body for 1 month. On investigation, her erythrocyte sedimentation rate was elevated (82 mm/h; range 0–20 mm/h) and the lymphocyte count was low, at 15% (range 25–40%). All other investigations for fever, including Widal test for typhoid, peripheral smear for malaria and urine routine and culture for urinary tract infection, and chest X-ray were negative. CT chest was ordered as further evaluation, which detected axillary lymphadenopathy. A biopsy from the axillary lymph node diagnosed her as a case of Hodgkin’s lymphoma (lymphocyte predominance). Her CT abdomen and pelvis showed hepatosplenomegaly with paraaortic, retrocaval and aortocaval lymphadenopathy. Her Lactate dehydrogenase LDH level was elevated to 554 IU/L (250–480). The bone marrow biopsy was negative for marrow infiltration by disease. Overall, she was staged as stage III Hodgkin’s disease.

She received a total of six cycles of standard chemotherapy with ABVD over 6 months. After completion of chemotherapy, she was referred to our department for response evaluation with FDG PET-CT. After routine clinical history and examination, she still had clinically palpable enlarged left axillary lymph node. She was injected 7.5 mCi of FDG IV and instructed to avoid talking and unnecessary body movements. Time of flight whole-body PET-CT scan was performed on a Philips Gemini PET-CT scanner 75 min after FDG injection. Contrast CT was performed for the purpose of attenuation correction and anatomical correlation. Her blood glucose level was 99 mg/ml prior to the scan.

Her scan showed an enlarged necrotic left axillary (7.2 cm×5.8cm×2.6 cm) and retrocardiac (2.2 cm×1.8 cm) nodes. Both these nodes did not show any significant FDG uptake. Diffusely increased FDG uptake was noted in the bone marrow, which was attributed to the post-chemotherapy bone marrow stimulation. Overall, the scan showed no evidence of demonstrable metabolically active disease suggesting good response to chemotherapy.

**Figure 1:**
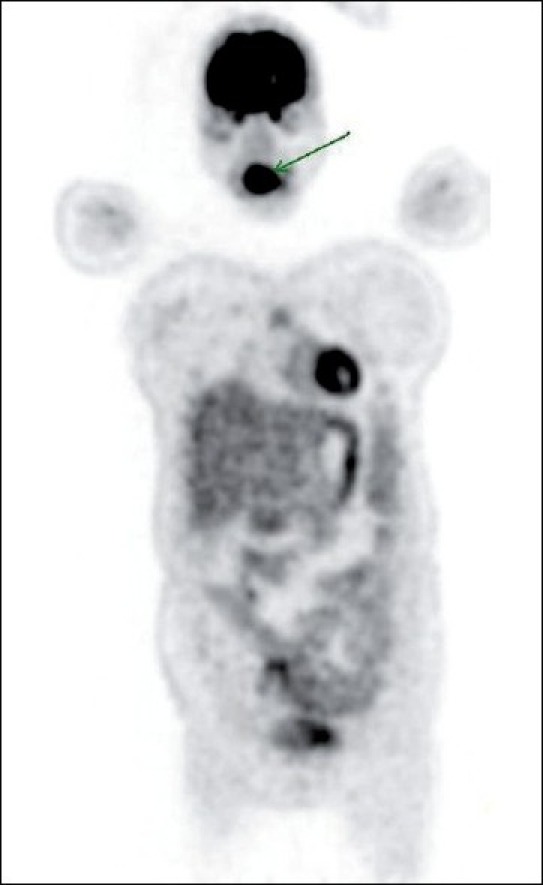
Coronal section of the whole-body positron emission tomography image showing intense fluorodeoxyglucose uptake in the region of the tongue

**Figure 2:**
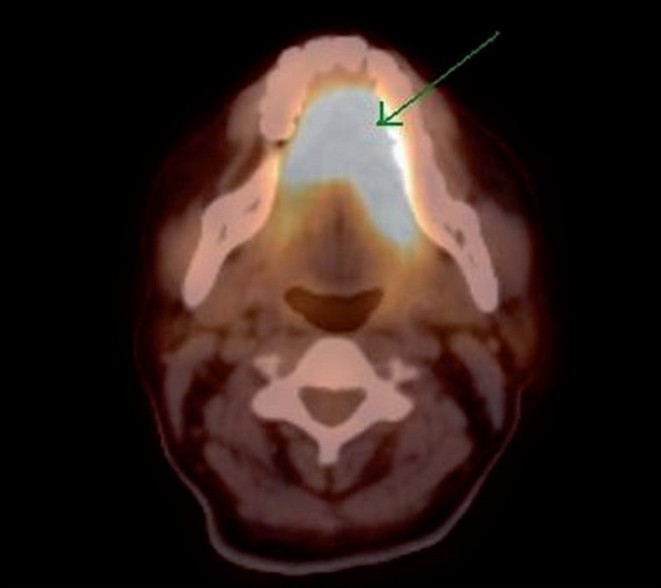
Transaxial section of the positron emission tomography-computed tomography fusion image showing intense fluorodeoxyglucose uptake in the anatomically normal tongue

**Figure 3:**
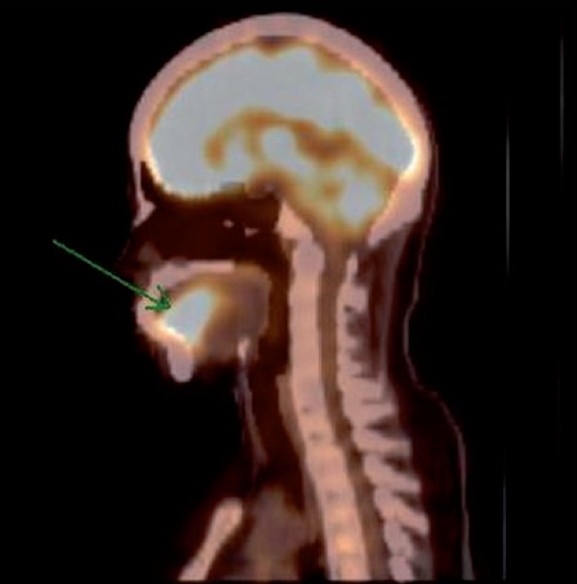
Saggital section of the positron emission tomography-computed tomography fusion image showing intense fluorodeoxyglucose uptake in the tongue

The interesting finding that was very striking was intense FDG uptake in the region of the oral cavity [Figure 1], which localized to the tongue on the CT scan. The tongue was of normal architecture on the CT scan [Figures2 and 3]. However, that did not explain the high maximum standardized uptake value [SUVmax] of 15.4 in the tongue. Hence, local examination of the tongue was carried out, which revealed mildly thickened tongue with involuntary tongue movements. On detailed questioning, the hidden story emerged as follows…

## DISCUSSION

The patient was mentally stable till she was diagnosed with lymphoma. However, after completion of the first cycle of chemotherapy, the patient was brought to the hospital by relatives with complaints of irrelevant talk, confused mental status, and depressed look. On psychiatric evaluation, she was diagnosed as a case of depression with acute anxiety and obsessive compulsive disorder. Her electro-encephalogram EEG and thyroid profile were normal. She was advised to take antipsychotic medication T. Clonazepam 0.25 mg ½-1/2-1 per day and T. Escitalopram 10 mg ½ per day for 1 week. She improved clinically and subsequently completed her chemotherapy cycles. On follow-up, the dose of Clonazepam was tapered to 0.25 mg 1 per day. Overall, she took Clonazepam for a period of 4 months before the scan and was on medication when the scan was performed.

On review of the literature, we found that Clonazepam is a commonly used antipsychotic medication that is a dopamine antagonist. As compared with older dopamine antagonists, it is rarely known to cause tardive dyskinesia.[1] In fact, it is one of the medications that is used for the treatment of tardive dyskinesia.[1,2] The exact etiology of tardive dyskinesia is not known. However, it is suggested that tardive dyskinesia may result primarily from neuroleptic-induced dopamine supersensitivity in the nigrostriatal pathway.[3]

Tardive dyskinesia involves involuntary movements of the tongue, lips, face, trunk, and extremities, which occurs in patients treated with long-term dopaminergic antagonist medications. Involuntary movements of the tongue are usually the first to be noticed. Although the exact incidence of this side-effect is not known, around 15–25% of the young adults treated for more than 1 year with older neuroleptic drugs may develop tardive dyskinesia. It is more likely to occur in older females. Also, the risk appears to be higher in patients with mood disorders.[4,5]

Therefore, in our patient, it was the repetitive involuntary movements of the tongue secondary to Clonazepam intake that caused an increase in the glucose metabolism by tongue muscles, which was demonstrated by intense FDG uptake on the scan. This case emphasizes the importance of being aware of false-positive findings that may occur due to the unique metabolic nature of FDG PET scanning. It also underlines the importance of detailed clinical history and examination.
